# Cyclic nucleotide signalling in kidney fibrosis

**DOI:** 10.3390/ijms16022320

**Published:** 2015-01-22

**Authors:** Elisabeth Schinner, Veronika Wetzl, Jens Schlossmann

**Affiliations:** Pharmacology and Toxicology, University Regensburg, Regensburg 93053, Germany; E-Mails: elisabeth.schinner@chemie.uni-regensburg.de (E.S.); Veronika.wetzl@chemie.uni-regensburg.de (V.W.)

**Keywords:** signalling, cyclic nucleotides, cyclic guanosine monophosphate, cyclic adenosine monophosphate, kidney fibrosis

## Abstract

Kidney fibrosis is an important factor for the progression of kidney diseases, e.g., diabetes mellitus induced kidney failure, glomerulosclerosis and nephritis resulting in chronic kidney disease or end-stage renal disease. Cyclic adenosine monophosphate (cAMP) and cyclic guanosine monophosphate (cGMP) were implicated to suppress several of the above mentioned renal diseases. In this review article, identified effects and mechanisms of cGMP and cAMP regarding renal fibrosis are summarized. These mechanisms include several signalling pathways of nitric oxide/ANP/guanylyl cyclases/cGMP-dependent protein kinase and cAMP/Epac/adenylyl cyclases/cAMP-dependent protein kinase. Furthermore, diverse possible drugs activating these pathways are discussed. From these diverse mechanisms it is expected that new pharmacological treatments will evolve for the therapy or even prevention of kidney failure.

## 1. Renal Fibrosis and Involved Signalling Pathways

Cyclic nucleotides cyclic adenosine monophosphate (cAMP) and cyclic guanosine monophosphate (cGMP) are important second messengers regulating chronic kidney disease. These signalling molecules act in a different manner upon various chronic kidney diseases (CKDs) which result from diverse causes. Disorders which often generate CKD comprise diabetic nephropathy, glomerulosclerosis and nephritis.

### 1.1. Fibrotic Kidney Diseases

Renal fibrosis is commonly found in CKD, e.g., diabectic nephropathy, glomerulosclerosis and lupus nephritis. These CKDs can be caused by oxidative stress, hypoxia, inflammation, autoimmune disease or altered metabolism. Acute insult of the kidney by ischemia or toxins can also finally result in CKD. However, there are common disease patterns in various CKDs, as the formation of myofibroblasts (which secrete in turn extracellular matrix (ECM) proteins) is regularly the first step in the progression of fibrosis. This leads to several renal damages including fibroblast activation, matrix deposition and inflammatory processes like interstitial leukocyte accumulation. The differentiation of progenitor cells, e.g., fibroblasts, pericytes, endothelial cells, epithelial tubular cells or bone-marrow derived fibrocytes into myofibroblasts is very important for the development of fibrosis. It is still not defined and highly discussed which cells are considered to be the primary source here [1,2,3,4]. For a long time, it was suggested that the differentiation of tubular cells into myofibroblast, the epithelial mesenchymal transition (EMT), is the main process for fibrosis induction. Meanwhile, it is thought that pericytes, interstitial fibroblasts and fibrocytes are major sources of this differentiation process [3,5]. In glomerulosclerosis, besides mesangial cells, also podocytes might be a source for pericyte differentiation into myofibroblasts [6]. The differentiation of progenitor cells is enhanced by profibrotic mediators, e.g., transforming growth factor β (TGFβ), connective tissue growth factor (CTGF) or Rho/Rho kinase [7]. The underlying signalling pathways are crucial for fibrotic progression. Therefore, the concentration or activity of these fibrotic markers is often determined to evaluate the development of fibrosis. Following differentiation, myofibroblasts lead to cytokine secretion, e.g., interleukin 1 (IL-1), tumor necrosis factor α (TNFα), platelet derived growth factor (PDGF), and enhanced ECM deposition of collagens or fibronectin. Consequently, the regulation of ECM degrading matrix metalloproteases (MMPs), e.g., MMP2 or MMP9, and their inhibitors TIMPs (tissue inhibitors of MMPs) is very critical for balancing the abundance of extracellular matrix proteins. Though, the MMP/TIMP ratio is often disturbed in fibrotic diseases. Moreover, inflammatory processes, e.g., activation of macrophages or T-lymphocytes, also influence fibrotic disease progression. Profibrotic cytokines, released by leukocytes induce oxidative stress and production of reactive oxygen species (ROS) [8,9]. Besides that, oxidative stress, e.g., caused by aldosterone, is involved in fibrotic disease progression [10]. Moreover, chronic hypoxia or stable expression of the hypoxia inducible factor 1α (HIF-1α) in tubuloepithelial cells promotes renal interstitial fibrosis [11]. A further factor for fibrosis progression might be altered metabolism by mitochondrial dysfunction and thereby generation of mitochondrial ROS [12,13].

Interference of profibrotic signalling pathways is a selective tool for disease suppression [14]. Inhibitors of TGFβ or of its signalling pathways, preventing the myofibroblast differentiation, are valuable as antifibrotic agents. Expression of TGFβ can be reduced e.g., by *pirfenidone*, which might be effective for treatment of diabetic kidney disease (Table 1). Additionally, it improves oxidative stress induced by mitochondrial dysfunction [15,16]. Signalling of cyclic nucleotides can act on several parts of these fibrotic processes as they suppress, e.g., interstitial fibrosis via reduced TGFβ signalling and myofibroblast formation or reduction of oxidative stress [10,17]. Details of these effects of cyclic nucleotides will be given in Section 2.1. and Section 2.2.

Diabetic nephropathy (DN) is the most important cause for end-stage renal disease and kidney failure. DN is often associated with hypertension and symptoms of DN are albuminuria, glomerulosclerosis and interstitial kidney fibrosis. Development of DN is strongly enhanced in endothelial nitric oxide synthase knockout mice (eNOS^−/−^), implicating an important function of this signalling defect in DN, most likely through hypertension [18,19]. Increases of the renin angiotensin aldosterone system (RAAS) are associated with diabetic nephropathy and fibrosis. Diverse signalling pathways including JAK/STAT and TGFβ are strongly activated by angiotensin II (ATII) leading to diabetic glomerular fibrosis and sclerosis [20]. Hence, treatments with RAAS inhibitors are a common therapy option of DN (Table 1) [21]. Remarkably, in a diabetic rat model an increase of aldosterone was concomitant with nitric oxide (NO)/cGMP decrease. The reduction of aldosterone by the renin inhibitor aliskiren or the calcium channel blocker amlodipine restored NO/cGMP levels [10]. Further possible treatments are tested regarding Rho kinase inhibition which experimentally improves glomerular haemodynamics in diabetic nephropathy [22]. Diminished blood pressure in hypertension confers protection against fibrosis. However, there are effects which are presumably independent of blood pressure reduction. The combined treatment with the angiotensin receptor blocker telmisartan and the sGC stimulator riociguat improved glomerular and interstitial fibrosis in comparison to telmisartan treatment alone without additional blood pressure reduction [23].

Glomerulosclerosis is frequently found in kidney diseases. A specific form, the idiopathic focal segmental glomeruloclerosis (FSGS), is a major cause of primary kidney disease resulting in nephrotic syndrome and end-stage renal disease [24]. FSGS causes podocyte injury which alters the permeability and selectivity of the glomerular barrier and leads to proteinuria [25,26]. The serum soluble urokinase receptor (suPAR) activating podocyte β3 integrin could be involved in induction of FSGS [27,28]. There are multiple secondary causes for this disease including diverse forms of glomerulonephritis, diabetes mellitus, arterial hypertension or Bartter syndrome. Current therapy options are glucocorticoids, calcineurin inhibitors, cytostatics and/or mycophenolate mofetil (Table 1, summarized in [26]). About half of the patients show total or strong reduction of proteinuria, whereas others are resistant to medication. Recently, rituximab—A monoclonal CD20 antibody which reduces circulation of B cells—Was clinically tested. Steroid-resistant FSGS showed no remission of the disease. However, other studies reported an improvement of FSGS upon rituximab or adrenocorticotropin (ACTH) treatment [25,29,30]. Still, new treatments for resistant FSGS are needed. In this regard, it might be interesting that cyclosporine is protective against FSGS via an increase in intracellular cAMP [31]. Therefore, enhancement of cAMP might be a valuable target for this disease. Furthermore, reduction of glomerulosclerosis by cGMP modulators is also common in renal fibrotic models (see below).

Lupus nephritis is a rare autoimmune disease leading to glomerulosclerosis, tubular atrophy and interstitial fibrosis that all result in renal failure. Enhanced cellular metabolism and hypertrophy was often observed [32]. The TNF-like weak inducer of apoptosis/Fibroblast growth factor-inducible 14 (TWEAK/Fn14) system is an inducing pathway in lupus nephritis and might be a pharmacological target for treatment of this disease [33]. A rodent model of systemic lupus is the MRL/lpr lupus prone mouse in which increased cGMP phosphodiesterase (PDE) and decreased cGMP levels was detected [34]. Actual common treatments of lupus nephritis or systemic lupus erythematodes (SLE) include broad spectrum steroids [35] or belimumab [36]. Besides that, cytostatics azathioprin or cyclophosphamide as well as hydroxychloroquine are used for treatment [37,38].

### 1.2. Renal Fibrotic Models

Transgenic or knockout animals, particularly mice, in combination with disease models for diverse renal fibrotic diseases are beneficial for understanding the diverse mechanisms in chronic kidney diseases. Knockout (KO) of genes in the AC/cAMP or natriuretic peptide/NO/cGMP signalling pathways (e.g., eNOS^−/−^, sGC^−/−^, GCA^−/−^ or PKG1^−/−^ mice) or overexpression of proteins (e.g., PKG transgenic mice) are important tools for studying the various diseases. The use of these mice for the analysis of renal fibrotic diseases will be presented in the diverse parts of this article (see chapter 2). siRNA is also used for examination of cellular signalling pathways, e.g., in Madin-Darby canine kidney epithelial (MDCK) or fibroblast cells.

Several models of diabetic nephropathy exist which are validated for the clinical features of human DN regarding e.g., decrease of kidney function, albuminuria and interstitial fibrosis [18]. However, none of the available models resemble all of these criteria. It is important to note that the induction of DN is dependent on the murine strain used. Furthermore, murine models often reveal only early stages of DN because the induction of interstitial fibrosis is less observed than in human DN. Common models for type 1 diabetes are mice injected with streptozotocin or genetic models e.g., OVE26 mice which carry a transgene overexpressing calmodulin in pancreatic β cells resulting in early onset of type I diabetes, in combination with unilateral nephrectomy [39]. For type 2 diabetes, e.g., ob/ob mice in combination with eNOS^−/−^ mice are used. Furthermore, hypertension e.g., induced with renin, *ATII or aldosterone* is an important factor enhancing DN and therefore to evaluate its renal effects [10,40]. Deoxycorticosterone acetate (DOCA)-salt hypertensive rats are often analyzed (DOCA and sodium chloride applied to uninephrectomised rats) which develop oxidative stress and inflammation. The effects of cGMP and cAMP modulators on diabetic nephropathy tested with these models will be discussed in the respective parts of Section 2.1.1. and Section 2.2.1.

Unilateral ureteral ligation (UUO) is a common surgery for the analysis of interstitial kidney fibrosis. This model is versatile for the elucidation of fibrotic disease mechanisms, resembles the various factors of interstitial fibrosis, is highly predictable [41] and often applied in the analysis of cyclic nucleotide signalling (see Section 2.2). The outcome is very rapid (between 3 and 14 days) and, therefore, it is discussed whether it features all phases of chronic kidney disease.

Renal ischemia/reperfusion is a preferred model for studying acute kidney injury [42]. It leads to lesions of tubular epithelial cells, inflammation and tubulointerstitial fibrosis. These responses are often not reversible and, therefore, might lead to CKD and kidney dysfunction. This model was used to study effects of tadalafil and CNP in acute kidney injury (see Section PDE Inhibitors and Natriuretic peptides).

Renal injury upon 5/6 nephrectomy is a valuable model for the analysis of mechanisms associated with renal dysfunction of the remnant kidney [41]. Apoptosis, inflammation and fibrosis via tubulointerstitial injury are main factors caused by renal ablation. Notably, there are differences in the responses of diverse murine strains. Furthermore, the analysis of the damaged tissue is limited by the small size of the remnant kidney. Several studies using this model revealed the suppressive effect of the PDE5 inhibitor sildenafil in fibrosis mechanisms (see Section PDE Inhibitors).

cAMP and cGMP are suppressive in several fibrotic diseases which will be explained explicitly in this review. The concentration of these cyclic nucleotides cAMP or cGMP is enhanced by adenylyl cyclases (AC) or guanylyl cyclases (GC), respectively, and modulated by several phosphodiesterases (PDEs). Examples regarding modulators of cyclic nucleotides cAMP or cGMP in renal fibrotic diseases and pharmacological treatments will be given in Section 2.1. and Section 2.2. of this article.

**Table 1:** Renal fibrotic diseases and its actual or clinically tested treatments.

Renal Fibrotic Disease	Causes	Profibrotic Signalling Pathways	Actual or Clinically Tested Treatments	Literature
Diabetic nephropathy	Hyperglykaemia; Hypertension	DM I, II; RAAS; JAK/STAT eNOS-dysfunction TGFβ	RAAS blockade Pirfenidone	[16,21]
Glomerulo-sclerosis (e.g., FSGS)	DN, Hypertension; Nephrotic syndrome; (FSGS)	e.g., DM I, II; RAAS; suPAR (FSGS)	RAAS blockade, Pirfenidone, FSGS: Glucocorticoids, Cytostatics, ACTH, Rituximab	[25,26,29,30,43]
Lupus nephritis	Autoimmune antibodies; Expansion of inflammatory cells [44]	TWEAK/Fn14	Steroids, Belimumab, Cytostatics: Azathioprin, Cyclophosphamide Hydroxychloroquine	[35,36,37,45]

DN: Diabetic nephropathy; DM: Diabetes mellitus; FSGS: Focal segmental glomeruloclerosis; RAAS: Renin angiotensin aldosterone system; suPAR: Serum soluble urokinase receptor; TWEAK/Fn14: TNF-like weak inducer of apoptosis/Fibroblast growth factor-inducible 14 system.

## 2. Cyclic Nucleotide Signalling Pathways and Their Potential as Therapeutic Options in Renal Fibrosis

Renal failure is a very common consequence of the above mentioned diseases. As the incidence of renal failure is rising worldwide, the prevention or delaying of renal dysfunction that leads to end-stage renal failure is the most important goal for pharmacological treatment of CKD [46]. Cyclic nucleotide modulation could be a therapeutic approach. This review focuses on the most relevant cyclic nucleotide signalling pathways in renal fibrosis as well as diverse drugs involved in cAMP or cGMP pathways which could be useful in the treatment of CKD.

### 2.1. Cyclic Adenosine Monophosphate (cAMP) Pathway

**Figure 1:**
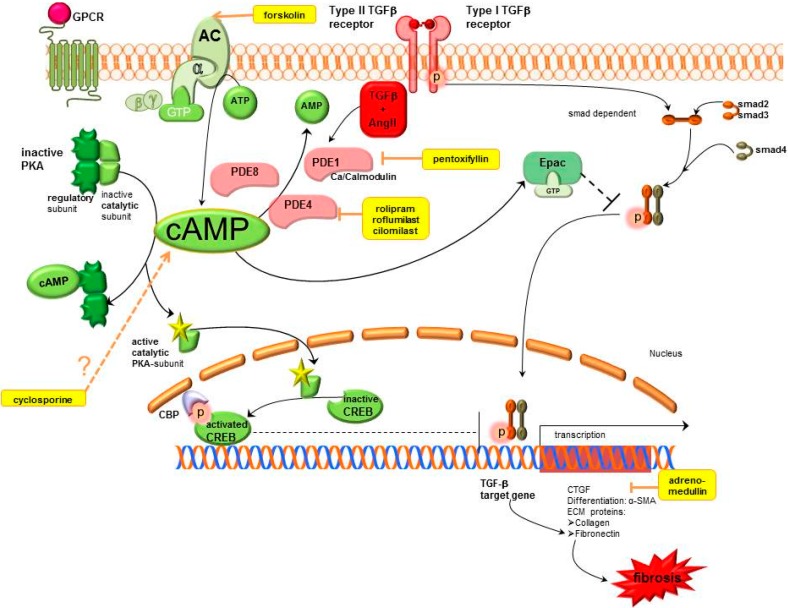
Cyclic adenosine monophosphate signalling pathways in kidney fibrosis including pharmacological treatment options. AC, adenylyl cyclase; AMP, adenosine monophosphate; ATP, adenosine triphosphate; cAMP, cyclic adenosine monophosphate; CBP, CREB binding protein; CREB, cAMP response element binding protein; CTGF, connective tissue growth factor; ECM, extracellular matrix; Epac, exchange protein directly activated by cAMP; GPCR, G protein coupled receptor; GTP, guanosine triphosphate; PDE, phosphodiesterase; PKA, protein kinase A; SMA, smooth muscle actin; smad, small mothers against decapentaplegic protein, TGFβ, transforming growth factor β.

The cAMP pathway exerts antifbrotic actions which include inhibition of EMT blockade of fibroblast proliferation and activation of the death of fibroblasts. These effects can arise in response to an increase in cAMP by AC activators, PDE inhibitors, cAMP analogues or pharmacological agents like Gs-linked G protein coupled receptors (GPCR) agonists and Gi-linked GPCR antagonists. Increased cAMP levels exert their effects through activation of protein kinase A (PKA) which is the classical signalling pathway. Thereby, cAMP binds to the regulatory subunit of PKA leading to dissociation of the catalytic subunit which subsequently phosphorylates target proteins. Stimulated PKA causes inter alia phosphorylation of cAMP response element binding (CREB) and subsequent CREB-mediated gene transcription. Detailled signalling mechanisms of cAMP are shown in Figure 1.

cAMP exerts antifibrotic effects in fibrosis, which are mediated by stimulation of PKA and activated CREB that thus blocks TGFβ mediated gene transcription. Furthermore, it activates Epac which blocks TGFβ mediated smad dependent gene transcription. Therapeutic options which enhance cAMP are AC stimulators, PDE inhibitors, cyclosporine and adrenomedullin (shown in yellow). Motifolio PPT Drawing Toolkit was used for designing the figure.

#### 2.1.1. cAMP Modulation

##### Adenylyl Cyclase (AC)

Adenylyl cyclases are enzymes which convert ATP into cAMP. At least nine isoforms of AC—AC1-9—and two splice variants of AC8 are known [47].

Previous studies have shown antifibrotic effects by an increase of AC5/6 expression in cardiac and pulmonary fibroblasts which prevents the differentiation of cardiac fibroblasts [48] and pulmonary fibroblasts to pathophysiological changed myofibroblasts [49]. This is not yet studied in renal fibroblasts, but AC5 expression was also shown in the kidney [50].

Forskolin is an AC activator which increases intracellular cAMP levels by activation of membrane bound AC. Clinical data about the usage of AC activators are not yet available. As many disorders, e.g., cardiovascular diseases, glaucoma, asthma *etc.*, are discussed for the application of forskolin, the risk-benefit ratio is not yet fully evaluated [51]. Nevertheless, antifibrotic effects of forskolin in renal mesangial cell cultures were already observed by reducing glomerular mesangial cell growth [52] or inhibiting *CTGF* gene expression by forskolin [53]. CTGF is a growth factor which is not present in healthy kidne but which is induced in fibrotic pathological condition, like glomerulosclerosis or diabetic nephropathy.

##### Phosphodiesterases (PDEs).

PDEs are enzymes which catalyze the hydrolysis of cAMP and/or cGMP, thereby, regulating the cAMP and cGMP levels [54]. The PDEs can be subdivided into at least 11 structurally related gene families (PDE 1 to 11) based on their ability to hydrolyze either preferentially cAMP, cGMP or both [55]. PDE1, PDE4 and PDE8 are important enzymes for cAMP signalling in the kidney. cAMP might be increased by PDE inhibitors.

PDE1: PDE1 consists of three genes—*PDE1A*, *B* and *C*—And belongs to the Ca^2+^ calmodulin-activated PDE family. Thereby, the activity of PDE1 family members can be increased up to tenfold in the presence of Ca^2+^ calmodulin [56,57]. PDE1A represents the predominant isoform but has a higher affinity for cGMP than for cAMP [57]. In cardiac fibroblasts, PDE1A is highly upregulated after stimulation with ATII and TGFβ [58]. Moreover, it is reported that the PDE1 inhibitor IC86340 decreased ATII or TGFβ induced cardiac myofibroblast activation, ECM production, and profibrotic gene expression. Thereby, PDE1 inhibition also mediates the antifbrotic effects via cAMP [58]. The PDE1 isozymes are abundant in the kidney and some isoforms of PDE1C exhibit high affinity for cAMP [59]. Thus, increased cAMP levels induced by specific PDE1 inhibitors could be beneficial in renal disease, but data about PDE1 inhibition in the kidney are lacking.

PDE4: Another PDE isoform, which specifically hydrolyses cAMP, is PDE4. Souness *et al.*, reported that inhibitors of PDE4 exert antiinflammatory effects by suppression of many inflammatory cell responses, TNFα release and ROS generation. Consequently, PDE4 inhibitors can be used in the therapy of different diseases characterized by excessive cytokine production [60]. In renal diseases protective effects—Including antifibrotic effects—Of PDE4 inhibition have already been investigated.

cAMP hydrolysis in the renal mesangial cells is particularly mediated via PDE3 and PDE4 [61]. Inhibition of PDE4 has repeatedly shown suppressive effects on TGFβ signalling which has relevant therapeutic effects on excessive tissue remodeling, e.g., glomerulopathies. PDE4 inhibitors decrease fibroblast activity through elevated cAMP, and thus PKA, by decreasing collagen contraction and fibroblast chemotaxis towards fibronectin, especially in the presence of TGFβ signalling *in vitro*. Moreover, PDE4 inhibitors normalize the release and activation of tissue-degrading MMPs in TNFα cultured fibroblasts [62,63,64]. In a rat glomerulonephritis model, improvement of renal function and renal structure was observed [65,66].

Roflumilast, a selective PDE4 inhibitor was tested in type1 diabetic nephropathy induced by streptozotocin. Treatment with roflumilast decreased oxidative stress as well as extracellular matrix proteins such as fibronectin, collagen and apoptosis which was demonstrated by the TUNEL assay. The antioxidant enzyme heme oxygenase-1 is elevated in type 1 diabetic kidney whereas FoxO1, a transcription factor involved in oxidative stress is decreased [67]. Roflumilast was able to reverse that pathological condition.

However, the above mentioned PDE4 inhibitors, e.g., roflumilast (see Section 2.1.2) have several side effects, like central nervous or gastrointestinal disturbances [68].

Recently, a selective PDE4 inhibitor TJN-598 was established which showed lower excretion of TGFβ as well as lower mesangial matrix index, but—Inconsistent with the commonly used PDE4 inhibitors—Could not improve protein excretion [69]. (The mesangial matrix index evaluates mesangial expansion by the ratio of mesangial area to total glomerular area, whereas mesangial expansion is a product of glomerular hypercellularity, widening as well as mesangial matrix accumulation.).

NCS613 preferentially inhibits PDE4C which prevents disease progression in a lupus nephritis disease experimental model by ameliorating proteinuria [70].

PDE8: PDE8 is a cAMP specific PDE [60]. Upon TGFβ stimulation cardiac myofibroblasts generate less cAMP compared with fibroblasts because the synthesis of cAMP by AC5/6 expression was down-regulated and the degradation of cAMP by the PDE8 isoform PDE8A was upregulated in myofibroblasts [71]. In the kidney, mRNA expression of AC5/6 [47] and PDE8A [72] was already shown. Therefore, a TGFβ induced downregulation of cAMP in renal myofibroblasts could also be conceivable, but it is not yet examined.

The methylxanthine derivate pentoxifylline is an unspecific PDE inhibitor which has antifibrotic and antiinflammatory properties in several models of disease and is used for peripheral vascular diseases in clinical practice [73].

Renal antifibrotic effects of this unspecific PDE inhibitor were described in several animal models. In an anti-Thy1 nephritic rat model, mRNA levels of type I, type III and type IV collagen as well as fibronectin were decreased in pentoxifylline treated rats compared to controls. Additionally, ICAM-1 and MCP-1 mRNA levels were reduced by pentoxifylline, in which cAMP involvement is supposed but evidence is lacking so far [74]. Similar antifibrotic results were observed in rats with 5/6 subtotal nephrectomy [75].

In tubulointerstitial fibrosis induced by unilateral ureteral obstruction, expression of remodeling biomarkers like Col1A1 and CTGF as well as α-smooth muscle actin (α-SMA), a marker for myofibroblast accumulation, are diminished by pentoxifylline which mediates its action via cAMP and subsequently PKA to abrogate TGFβ signalling [76]. In a similar model, reduction of total volume of interstitial fibrosis was observed as well [77].

Remodeling processes—Particularly renal scarring—Are also a consequence of pyelonephritis. Pentoxifylline prevented renal scar formation after induction of pyelonephritis in an experimental rat model when antibiotic therapy was delayed [78].

The renoprotective effect of pentoxifylline was already shown in the PREDIAN clinical trial, in which patients with type 2 diabetes mellitus and chronic kidney disease had a lower decline in estimated glomerular filtration rate with pentoxifylline treatment in addition to RAAS blockade [79]. These results indicate that multiple inhibitors of PDE isoforms are potential targets in the therapy of renal fibrosis. Inhibitors of PDE4, which specifically prevent degradation of cAMP, are in clinical development.

#### 2.1.2. cAMP Effectors

##### Protein Kinase A-cAMP Response Element Binding (PKA-CREB)

In human epidermal HaCat keratinocytes, it was shown that cAMP elevating agents such as the AC activator forskolin prevent TGFβ2 signalling via PKA [80]. TGFβ transduces intracellular signals through type1 (TGFβ-R1) and type2 (TGFβ-R2) receptors. Receptor associated smad (R-smads) proteins, such as smad2 and smad3 are phosphorylated and activated by type1 receptors of TGFβ. Upon phosphorylation, the R-smads build a complex with smad4 which is a common mediator for all receptor activated smads. R-smad/smad4 complexes are then translocated into the nucleus where they modulate the transcription of many genes [81,82]. However, the cAMP/PKA/cAMP response element binding (CREB) cascade blocks TGFβ specific smad-dependent transcription. cAMP elevating agents abolished interactions of the TGFβ2 induced smad3/4 complex with the transcription co-activators CREB protein in a PKA dependent manner. Thereby, the smad translocation into the nucleus in response to TGFβ was not affected by cAMP in the examined HaCat keratinocytes [80]. These results are in accordance with Lin *et al.*, that increased cAMP accompanied by PKA induced CREB phosphorylation attenuated renal tubulointerstitial fibrosis. Thereby, increased cAMP levels were achieved by pentoxifylline—A general inhibitor of cAMP dependent PDEs—Which leads to a block of TGFβ induced smad3/4 dependent gene transcription. In this study, the inhibition of the profibrogenic CTGF was shown. However, smad activation and nuclear translocation were also not affected by cAMP/PKA [76].

CREB effects were shown by modulating cAMP via pentoxifylline or forskolin as direct modulators of CREB are lacking.

##### Exchange Protein Directly Activated by cAMP (Epac)

cAMP is able to stimulate PKA-independent Epac which is a guanine nucleotide exchange protein for the small GTPase Rap1. Activation of Epac by cAMP leads to release of the guanine nucleotide GDP and binding to GTP [83]. Epac regulates different functions like migration, proliferation and apoptosis via Rap1 [84]. It was shown that fibrosis inhibits Epac expression. Therefore, activation of Epac acts antifibrotically by inhibition of collagen type I- and collagen type III expression. Moreover, Epac interacts with TGFβ-R1 resulting in inhibition of phosphorylation of smad2 and transcriptional activation [85]. The importance of the cAMP-Epac-Rap signalling for modulating myofibroblast stimulation and ECM production was also described in the heart by Miller *et al.* [58].

A model to study EMT, which is a mechanism of tissue fibrosis, is the use of renal epithelial cells such as MDCK cells treated with TGFβ. MDCK cells base on epithelial phenotype with high E-cadherin expression and low α-SMA expression. TGFβ induces EMT by increasing α-SMA (and proteins characteristic of fibroblasts such as collagens) and decreasing E-cadherin expression (and other proteins characteristic of epithelial cells). Treating these MDCK cells with cAMP-derivative inhibits the upregulation of α-SMA via Epac. Therefore, Epac acts antifibrotically via inhibition of profibrotic TGFβ signalling [86].

A recent study by Stolman *et al.*, demonstrated that Epac/Rap stimulation ROS production in the kidney [87]. Uncontrolled production of ROS mediated cellular injury and also occured during renal fibrosis [88].

Thus, activation of Epac/Rap signalling may protect against renal fibrosis, but data about pharmacological modulation of kidney fibrosis are lacking.

#### 2.1.3. Further cAMP Influencing Systems

Adrenomedullin particularly is a vasodilatory agent which was demonstrated to augment NO. Its antifibrotic effects are mediated through cAMP-mediated decrease of CTGF induction and Erk phosphorylation in renal interstitial fibrosis [89]. The renoprotective effects were observed in diverse hypertensive models, e.g., in Dahl salt-sensitive rats or in the DOCA-salt model [90,91].

Cyclosporine is widely used for the treatment of FSGS. Its renoprotective effects are partly mediated through hemodynamic effects by decreasing glomerular perfusion rate and by decreasing intracellular pressure, but also through reduction of T cell mediated cytokines. Presumably, an increase in glomerular cAMP levels following cyclosporine treatment is responsible for the improved permeability characteristics of the glomerular filtration barrier [31].

### 2.2. Cyclic Guanosine Monophosphate (cGMP) Pathway

Chronic organ injury, particularly kidney fibrosis, degrades NO producing cells, such as endothelial cells. The decreased availability of NO leads to reduced cGMP levels. Many studies report that enhanced cGMP levels have an effective antifibrotic benefit in various organs including the kidney [17].

**Figure 2:**
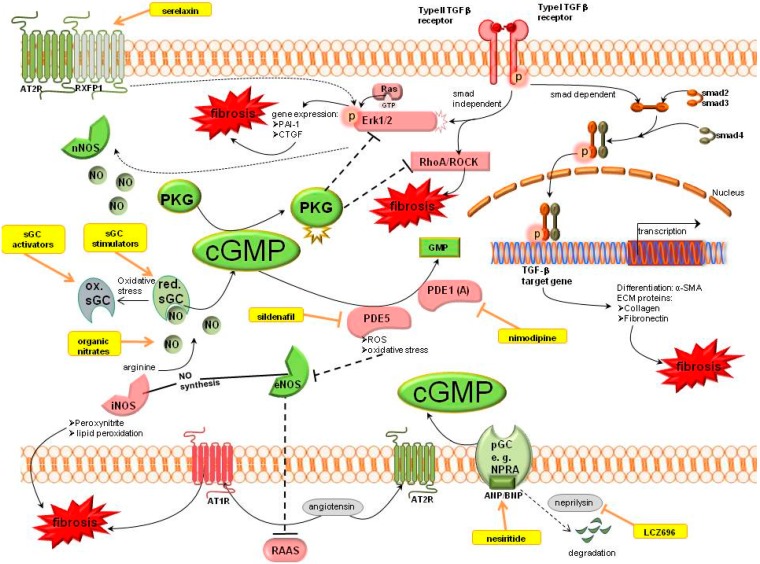
Cyclic guanosine monophosphate signalling pathways in kidney fibrosis including pharmacological treatment options. ANP, atrial natriuretic peptide; AT_1_R, angiotensin II receptor type 1; AT_2_R, angiotensin II receptor type 2; BNP, brain natriuretic peptide; cGMP, cyclic guanosine monophosphate; CNP-c, type natriuretic peptide; CTGF, connective tissue growth factor; ECM, extracellular matrix; eNOS, endothelial NO synthase; ERK1/2, extracellular-signal regulated kinase; GMP, guanosine monophosphate; GTP, guanosine triphosphate; iNOS, inducible NO synthase; nNOS, neuronal NO synthase; NO, nitric oxide; NPRA, natriuretic peptide receptor A; PAI-1, plasminogen activator inhibitor 1; PDE, phosphodiesterase; pGC, particulate guanylyl cyclase; PKG, cGMP dependent protein kinases; RAAS, renin angiotensin aldosterone system; Ras, Rat sarcoma protein, RhoA, particular Rho protein; ROCK, Rho associated protein kinase; ROS, reactive oxygen species; RXFP1, relaxin family peptide receptor 1; sGC, soluble guanylyl cyclase; SMA, smooth muscle actin; smad, small mothers against decapentaplegic protein; TGFβ, transforming growth factor β.

Nitric oxide synthases (NOS) produce NO which activates the soluble guanylyl cyclase (sGC). sGC is the NO receptor mediating the downstream signalling by the generation of cGMP. Increased cGMP levels lead to activation of cGMP dependent protein kinases (PKG). cGMP can also be produced by natriuretic peptides (ANP/BNP) which stimulate the particulate guanylyl cyclase (pGC). The degradation of cGMP is mediated by PDEs. Detailled signalling of cGMP is shown in Figure 2.

cGMP mediates its antifibrotic action (shown in green) via activation of PKG which is able to inhibit the profibrotic TGFβ signalling (shown in red). TGFβ signalling is mediated via a smad dependent pathway to increase target gene transcription, or a smad independent pathway which activates Erk1/2 and RhoA/ROCK signalling. Therapeutic options which enhance cGMP are serelaxin, sGC modulation, organic nitrates, PDE inhibitors or natriuretic peptide analogues or their modulators (shown in yellow). Motifolio PPT Drawing Toolkit was used for designing the figure.

Some treatment options are in development for the augmentation of the cGMP pool [92]. The aim of these agents is to ameliorate or prevent the progression of fibrotic tissue.

#### 2.2.1. cGMP Modulation

##### Organic Nitrates

Organic nitrates are used for the treatment of cardiovascular disease for the last centuries, as they increase NO availability and thereby support the NO/cGMP signalling pathway. However, this therapy option is limited due to formation of nitrate tolerance as well as accumulation of ROS (e.g., peroxynitrite) under oxidative stress conditions. Beneficial effects in kidney fibrosis were shown in different glomerulonephritis experimental models after administering l-arginine supplements [93,94].

##### Nitric Oxide Synthase

The generation of NO is catalyzed by three NOS isoforms: type I (neuronal or nNOS), type II (inducible or iNOS) and type III (endothelial or eNOS). All three NOS isoforms are found in the kidney, but the expression of iNOS is more variable [95]. Furthermore, the contribution of eNOS, nNOS and iNOS to renal injury is very different.

eNOS: The eNOS activity is altered in diabetes and associated with the development of nephropathy in type1 and type2 diabetes patients [96]. Diabetic patients often develop nephropathy despite optimal therapy including adjustment of blood glucose. Moreover, superoxide producing enzymes promote an increased formation of ROS and eNOS uncoupling leading to decreased NO bioavailability [97]. Oxidative stress inactivates NO. Thus, reduced NO bioavailability accompanies chronic renal diseases [98]. Comparable results reported Liu *et al.*, that the activity of eNOS was decreased by the fibrotic process in the lung [99]. In the cirrhotic liver, cGMP synthesis was also reduced due to the declined activity of eNOS [100].

Moreover, eNOS counterbalances the activity of the RAAS. Thus, inhibition of NOS increases the activity of the RAAS resulting in augmented expression of fibrosis marker such as fibronectin and α-SMA [101]. Moreover, deletion or reduction of eNOS in animals leads to glomerular abnormalities and tubular cell death. eNOS deletion is realized in eNOS knockout models whereas eNOS reduction is, for example, realized by models of hypertension. Consequently, eNOS protects against renal injury [95,102].

nNOS: In contrast to eNOS, an increase in nNOS facilitates renal injury. It is speculated that increased nNOS expression promotes generation of oxidative stress and formation of ROS within the kidney. Therefore, inhibition of nNOS showed renoprotective effects. Thereby, the inflammatory cells and the number of CD68 positive cells were reduced [95].

iNOS: iNOS produces—In comparison to eNOS and nNOS—large amounts of NO that, under oxidative stress conditions, can form peroxynitrite. Peroxynitrite initiates lipid peroxidation, oxidative protein and DNA modifications [103]. Thus, the downregulation of iNOS expression can reduce toxic peroxynitrite reactions.

Jeong *et al.*, also proposed that decrease of iNOS could induce renoprotective effects in streptozotocin induced diabetic rats [104].

##### Soluble Guanylyl Cyclase (sGC)

Guanylyl cyclases are enzymes that convert guanosine triphosphate (GTP) to cGMP. Both types of guanylyl cyclases, particulate guanylyl cyclase (pGC) activated by atrial (ANP) and brain natriuretic peptides (BNP), and soluble guanylyl cyclase (sGC) stimulated by nitric oxide, can reduce cardiac fibrosis by increasing intracellular cGMP levels [105]. Furthermore, it is reported that NO via cGMP downregulates CTGF in rat mesangial cells. Thereby, NO activates the soluble form of guanylyl cyclase which leads to increased cGMP levels [106].

sGC modulation could be achieved by either sGC stimulators or sGC activators.

The target of sGC stimulator is the NO sensitive reduced Fe^2+^ redox state (NO dependent) whereas the target of sGC activator is the NO insensitive oxidized (Fe^3+^) sGC [107]. sGC stimulators bind NO-independently to a regulatory site of the α-subunit of sGC to stimulate the enzyme. Furthermore, they cause sGC to be more sensitive to endogenous NO.

If NO deficiency is the major issue, the use of sGC stimulators is suitable sensitizing sGC for NO. However, if heme oxidation or heme-free form of sGC is the major cause of disease, sGC activators are an appropriate tool for therapeutic use [97]. sGC activators bind to inactive sGC to additionally increase enzyme activity, when the prosthetic heme-group is oxidized or lacking. Activators might be introduced as therapy option particularly for pathological changes due to oxidative stress [108].

Thereby, it is important to know that oxidative stress, which is an accompanying effect of fibrosis, favours heme oxidation or heme-free sGC. Both sGC stimulators and sGC activators were used in studies for the treatment of kidney diseases. It is suggested that sGC modulators have direct antifibrotic actions that are presumably associated with enhanced NO/sGC/cGMP signalling [108,109,110,111].

sGC Stimulators: It is manyfold reported that the sGC-cGMP axis could be a therapeutic target of fibrosis of various organs. Stimulation of sGC decreased TGFβ induced collagen release by inhibition of Erk1/2 phosphorylation in human fibroblasts. However, nuclear p-smad2 and 3 levels, smad reporter activity and transcription of TGFβ target genes were unaffected by sGC stimulation. In murine sGC KO fibroblasts sGC stimulation showed no effects [112].

YC-1 is a benzylindazole derivative which has multiple actions—cGMP-dependent [113] or -independent [114] effects. cGMP dependent actions are, among others, inhibition of phosphodiesterase activity or sGC modulation [113]. It firstly provided sGC modulation, independent of NO bioavailability [115,116,117].

Antifibrotic effects of YC-1 were observed in diverse tissues, e.g., in a preventive model of hypoxia-induced pulmonary arterial hypertension (PAH), in which YC-1 alleviates right ventricular hypertrophy and pulmonary vascular remodeling [118].

cGMP-dependent antifibrotic effects of YC-1 via PKG1 in the kidney were described by Schinner *et al.* [119] in an animal model of interstitial kidney fibrosis induced by unilateral ureteral obstruction. YC-1 inhibited the profibrotic RhoA/ROCK pathway by the cGKIα isozyme, which blocked RhoA phosphorylation. As a consequence TGFβ stimulation as well as myofibroblast formation was suppressed. Moreover, the antifibrotic effect of YC-1 was boosted via HIF-1α inhibition in renal kidney fibrosis [11]. However, due to its cGMP-independent effects YC-1 is not applicable for clinical drug testing.

BAY 41-8543 and BAY 41-2272 are pyrazolopyridines which were developed from the structure of YC-1.

BAY 41-2272 attenuated remodeling and limited progression of fibrosis in an anti-Thy1 induced model of progressive kidney disorders, particularly in interstitial fibrosis [17,120]. Additionally, it reduced mesangial proliferation, matrix expansion and proteinuria in a rat model of mesangial proliferative glomerulonephritis compared to placebo [94,121].

BAY 41-8543 showed also a renal protective effect [122]. It restored or preserved renal structure and function in case of obstructive kidney disease by positively influencing α-SMA expression, collagen IV deposition and TGFβ1 mRNA expression [123].

The majority of available research is preclinical. Clinical experiments are limited due to unfavourable pharmacokinetic profile, CYP inhibition or induction and blood pressure decrease in healthy volunteers [124].

Riociguat (Bay 63-2521) is a further sGC stimulator causing antifibrotic effects in the kidney. Riociguat has two benefits—it sensitizes sGC to NO and can also increase sGC activity in the absence of NO. Riociguat (BAY 63-2521) is structurally similar to BAY41-8543 and the single sGC stimulator which has an acceptable oral bioavailability and thus, was successfully completing the clinical development program due to its pharmacokinetic profile. It was approved by health authorities for PAH and chronic thromboembolic pulmonary hypertension (CTEPH) [125]. Its tradename is Adempas^®^.

Administration of riociguat alone as well as in combination with the AT_1_R inhibitor telmisartan attenuated progression of renal fibrosis, especially diabetic nephropathy. Thereby the systemic inflammation was decreased which was measured by plasma TNFα levels [23]. In a similar fashion renal fibrotic tissue remodeling was markedly improved in a rodent model of pressure and volume overload by decreasing protein und mRNA expression of profibrotic osteopontin, TIMP1 and PAI-1 (plasminogen activator inhibitor 1) in the renal cortex [126].

Furthermore, in two independent models of hypertension, it was shown a potent protection against renal interstitial fibrosis and partially against glomerulosclerosis [127].

sGC activators: The sGC activators cinaciguat [128] and ataciguat (HMR1766) [129] have antiremodeling effects in the kidney.

Cinaciguat is in the clinical development program for acute heart failure, but in a high dose cinaciguat therapy (>200 µg/h) hypotension occurred as adverse event and subsequently the phase IIb clinical trial was stopped [130].

The NO independent sGC activator cinaciguat (Bay 58-2667) is characterized by activating sGC after oxidation of its hem group. Chronic renal failure is accompanied by impaired NO availability because the generation of NO is decreased, and NO is inactivated by ROS or its physiological action is impeded by dysfunctional sGC. The reduced NO availability promotes renal disease.

Cinaciguat attenuated remodeling and limited progression of fibrosis in models of pulmonary hypertension partially associated with eNOS-dependent generation of nitric oxide [110]. Furthermore, rats with subtotal 5/6 nephrectomy which were treated with cinaciguat had slowing renal disease progression, reduced left ventricular hypertrophy and preserved renal function by targeting oxidized sGC and increasing intracellular cGMP [128].

Beneficial effects of ataciguat (HMR1766) on structural parameters of renal damage and urinary albumin excretion in a remnant kidney model were demonstrated [129]. HMR1766 has blood-pressure-independent and sustainable antifibrotic effects.

sGC activators are promising compounds for the treatment of kidney fibrosis. Data about antifibrotic effects in the kidney are still to be fully elucidated.

##### PDE Inhibitors

PDE1: As already mentioned, PDE1 consists of three genes—*PDE1A*, *B* and *C*. PDE1A represents the predominant isoform which has a higher affinity for cGMP than for cAMP [57]. Inhibition of PDE1 by IC86340 reduced ATII or TGFβ induced activation of cardiac myofibroblasts, synthesis of ECM and expression of profibrotic genes. Thereby, the antifbrotic effects of this PDE1 inhibitor were also mediated via cGMP/PKG.

The phosphodiesterase 1 inhibitor nimodipine was investigated as new therapeutic strategy for the autoimmune disease systemic lupus. In MRL/lpr lupus-prone mice PDE1 activity was elevated in the kidney, which was accompanied by a decrease in cGMP levels. Treatment with nimodipine resulted in improvement of organ remodeling, especially kidney remodeling, as well as in reduction of hypercellularity [131].

PDE5: The cGMP pool is rapidly degraded by PDEs. PDE5 is the PDE isozyme which specifically hydrolyzes cGMP. At the renal level of rat kidney, PDE5 is expressed in inner medullary collecting duct cells, cortical tubules, mesangial cells, the vasculature and glomeruli. PDE5 inhibitors, including sildenafil and tadalafil, possess antiapoptotic and antioxidant properties. Consequently, they exert nephroprotective effects.

In lung fibrosis, the PDE5 inhibitor (KMUP-1) was able to reduce the fibrotic process. In this study, many possible mechanisms, which mediate the effects of PDE5 inhibition, were shown. Administration of KMUP-1 significantly attenuated the expression of active MMP2 which is upregulated in fibrosis. Moreover, TGFβ and CTGF expression was reduced by KMUP-1. Moreover, the TGFβ induced increase of the p-smad3/smad3 ratio was downregulated by administration of KMUP-1. Furthermore, KMUP-1 inhibited the profibrotic RhoA/ROCK signalling and enhanced eNOS activity which was decreased in fibrosis [99]. These antifibrotic mechanisms of PDE5 inhibition are not yet studied in the kidney although PDE5 inhibitors such as sildenafil and tadalafil are tested in renal diseases. Thus, these mechanisms and signalling pathways in lung fibrosis could also be an approach in renal fibrosis. The PDE5 inhibitor sildenafil was already tested many times regarding kidney function. In a mouse model of interstitial kidney fibrosis induced by UUO renal fibrosis was reduced after administration of sildenafil for 14 days. Amelioration of kidney fibrosis was perceived by increased cGMP levels, mediated partly through regulation of macrophages and tubular cells. This was associated with reduced renal TGFβ/smad signalling and decreased macrophages infiltration. Kidney fibrosis was evaluated via protein and mRNA expression of collagen type I, collagen type III as well as α-SMA [132]. The PDE5 inhibitor sildenafil was administered to rats with 5/6 nephrectomy immediately after renal ablation for 8 weeks. In this setting sildenafil prevented histological damage, inflammation and apoptosis leading to reduced worsening of renal function, ameliorated proteinuria and decreased hypertension. Simultaneously, urinary cGMP excretion was elevated with sildenafil treatment [133]. It additionally suppressed renal arteriolar remodeling as a reason for improved function of the remnant kidney [134]. DOCA-salt hypertensive rats develop renal dysfunction due to fibrotic remodeling processes. In this experiment sildenafil reduced remodeling biomarkers, e.g., α-SMA and fibronectin, and thereby prevented progression of tubulointerstitial fibrosis and glomerulosclerosis [135].

Another study found that sildenafil attenuates infiltration of macrophages (ED1 positive cells) reducing the production of inflammatory stimuli. Moreover, PDE5 inhibition prevented oxidative stress in diabetic nephropathy. Jeong *et al.*, speculated that decrease of iNOS by sildenafil could induce this renoprotective effect [104]. The inhibition of oxidative stress by sildenafil—Determinated by suppression of NADPH oxidase expression and therefore of superoxide formation—Parallels the results of Muzaffar *et al.* [136].

In the study of Sohotnik *et al.*, the PDE5 inhibitor tadalafil prevented kidney dysfunction and structural renal damage in an experimental model of renal ischemia-reperfusion (I/R) [137,138]. Tadalafil also reduced the urinary excretion of NGAL and KIM-1, which are two biomarkers indicating acute kidney injury.

##### Relaxin

The pregnancy hormone relaxin—Human relaxin II—Has shown antifibrotic effects in several experiments *in vitro* and *in vivo*. Its antifibrotic actions were demonstrated in several cell types, tissues, and organs, including lung, liver, heart, and kidney. The relaxin family peptide receptor 1 (RXFP1)—The most relevant receptor for relaxin’s antifibrotic effects—was already localized in different tissues, including the kidney [139,140].

Relaxin is a hormone that both reduces ECM production and increases its clearance. This was first found in 1929 by Hisaw *et al.*, who showed, that relaxin induces relaxation of the pelvic ligament as the ligament reveales remodeling of the collagen from dense bundles to looser, less structured fibers. In pregnancy, relaxin is responsible for widespread extracellular matrix remodeling in the cervix, vagina and in some species in the pubic symphysis [141].

The antifibrotic signalling mechanism of relaxin is not clearly understood so far and therefore, it has to be fully elucidated in the future. But a lot of research is done to generate a hypothesis for relaxin’s antifibrotic pathway. Relaxin is supposed to mediate its antifibrotic effect in renal myofibroblasts via the RXFP1 and a subsequent Erk1/2 phosphorylation which activates the nNOS/NO/cGMP-dependent pathway. The disruption of TGFβ signalling by relaxin was demonstrated by decreased ECM production and decreased myofibroblast differentiation in humans [142] as well as in rodents [143,144]. Additionally, relaxin leads to upregulation of MMPs—ECM degrading enzymes—And downregulation of TIMPs—Inhibiting enzymes of MMPs—Which is likely mediated through iNOS. This signalling mechanism is increased by blocking the TGFβ1/Smad2 signalling [145]. Current research indicated that relaxin signals through RXFP1-AT_2_R heterodimer complexes which are formed between RXFP1 and AT_2_R independent of ligand binding [146], which could explain relaxin’s antifibrotic effects especially in pathological conditions. In the lung, iNOS-NO-cGMP-PKG signalling was demonstrated to inhibit the profibrotic RhoA/ROCK which thus abrogated myofibroblast contractility [147]. Furthermore, eNOS appeared to be more relevant in vasodilating effects of relaxin [148]. This leads to the assumption that relaxin can act on the various NOS isoforms which depends on specific actions and tissues.

Relaxin deficient mice spontaneously develop fibrotic tissue in different organs, including the kidney [149]. They are associated with age-related fibrosis going along with renal hypertrophy, increase in total collagen content, interstitial fibrosis, glomerular sclerosis and a decline in renal function [150,151].

The exogenous infusion of serelaxin led to ameliorated progression of interstitial and glomerular fibrosis in several experimental models of renal diseases.

In aging rats the infusion of relaxin increased glomerular filtration rate as well as renal plasma flow and decreases renal vascular resistance, acutely caused by increased gelatinase activity [152]. Renal fibrosis caused by antiglomerular basement membrane disease decreased glomerulosclerosis and interstitial fibrosis [153]. ATII-induced hypertension preserved glomerular structure as well as reduced oxidative stress after a two week period of relaxin treatment [154].

In another animal model relaxin administration was started one week after induction of renal papillary necrosis by bromoethylamin injection for the duration of 28 days. Initial papillary necrosis results in interstitial fibrosis and renal insufficiency. Relaxin improved renal function by restoration of glomerular filtration rate indicated by reduced TGFβ, macrophage infiltration and reducing fractional area of interstitial collagen staining by 75% [155]. Relaxin was administered for the same duration in models of renal mass reduction [156] and for shorter [157] or longer duration [148] in hypertension which demonstrated decreased interstitial and glomerular fibrosis and normalized collagen accumulation in the kidney. Influence on reducing blood pressure was shown, but matchable results were achieved in blood pressure independent models [155].

In contrast, in ATII-induced organ damage [158] as well as in diabetic renal disease [159] relaxin could not reverse fibrotic processes.

Recently, a novel peptide was identified, CGEN25009, which has relaxin-like activity. Its action is mediated through RXFP1 receptor, involvement of cyclic nucleotides was observed in the antifibrotic mode of action [160]. Data about renal fibrosis are still lacking.

##### Natriuretic Peptides

Natriuretic peptides are divided into three peptides—Atrial (ANP), brain (BNP) and C-type (CNP) natriuretic peptides [161]. Biological action of ANP and BNP is mediated through binding to NPR-A to induce vasorelaxing, diuretic, natriuretic, antiproliferative, antihypertrophic and antialdosterone effects. NPR-B is the receptor for CNP to cause vasorelaxing and antifibrotic effects as well as playing a role in bone growth regulation and reproduction. Due to its paracrine and autocrine effects CNP as well as its receptor is located in several tissues, including the kidney [162].

It is reported that ANP/cGMP/PKG can abolish TGFβ induced nuclear translocation of p-smad2 and p-smad3 in rat pulmonary arterial smooth muscle cells. However, the phosphorylation of smad2/3 is not influenced by ANP/cGMP. It is discussed that cGMP/PKG phosphorylates smad2 or smad3 on additional serine residues which are different from the TGFβ phosphorylation sites leading to inhibition of nuclear translocation [163]. These results are in agreement with studies in cardiac fibroblasts. Here, the TGFβ induced nuclear translocation of p-smad3 was also abolished by cGMP. As already mentioned, PKG1 phosphorylated smad3, but at sites different from those required for its TGFβ induced nuclear translocation. Furthermore, the stimulation of the cGMP/PKG pathway by ANP reduced TGFβ induced myofibroblast differentiation, proliferation, collagen production and PAI-1 expression [164].

Thus the pharmacological potentiation of endogenous ANP or BNP may be a therapeutic approach for the treatment of renal fibrosis.

Disruption of the gene encoding for GC-A/NPR-A leads to the development of renal fibrosis. Thereby, TNFα and IL-6 are increased [165]. These findings are in accordance with Kumar *et al.*, who showed that increased renal NPR-A/cGMP signalling attenuates renal fibrosis [166]. ANP is a member of a natriuretic peptide family, which counterregulates renal hypoxia and the consequent process of fibrosis, exerting protective effects in response to oxidative stress and fibrosis [167]. An earlier study also demonstrated that activation of the endogenous NPR-A system and administration of ANP increased the cGMP levels leading to reduced renal interstitial fibrosis induced by UUO [168].

Nesiritide is the recombinant form of naturally occurring brain natriuretic peptide (BNP) enhancing cGMP levels via GC-A/NPR-A. This signalling pathway has demonstrated cardiac antihypertrophic and antifibrotic effects in several experiments.

In mutant mice lacking *Npr1* gene, which is encoding for GC-A/NPR-A, increased fibrosis, hypertrophic growth and remodeling of the kidney was observed [165].

Chronic excess of BNP has shown to ameliorate glomerular hypertrophy and mesangial expansion after renal ablation, improves immune-mediated renal injury and prevents glomerular injury in progression of diabetic nephropathy [169,170,171].

In the last years new chimeric natriuretic peptides were developed to generate more potent peptides. CBA-NP is a fusion of diverse amino acid sequences of ANP, BNP and CNP. CD-NP is a composition of CNP and DNP, which enables the newly created peptide to act on both natriuretic peptide receptors NPR-A and NPR-B. Both CD-NP and CBA-NP attenuated renal fibrosis resulting in an improved renal function [172,173].

CNP could be a novel approach for the prevention of acute kidney impairment after ischemia-reperfusion injury. Jin *et al.* demonstrated improved renal vascular function and degraded glomerular and tubular microstructure in an experimental rat model of ischemia reperfusion injury. This effect is supposed to be mediated via cGMP signalling [162].

These observations suggest that natriuretic peptides and their derivatives play a pivotal role in the renal antifibrotic properties. However, nesiritide could not provide improvement of kidney function in the ASCEND-HF clinical trial in acute decompensated heart failure [174].

LCZ696 is the first compound of a new drug class, ARNI (angiotensin receptor neprilysin inhibitor) and is now being investigated in patients with chronic kidney disease [175]. LCZ696 simulaneously blocks AT_1_R and neprilysin, which is a natriuretic peptide degrading enzyme, resulting in enhanced activity of natriuretic peptides. Recently, clinical data are promising for chronic heart failure patients with reduced ejection fraction as they are superior to angiotensin-converting enzyme (ACE) inhibition by reducing risk of death and hospitalization in heart failure [176]. LCZ696 may provide kidney protection through blocking the profibrotic RAAS and concomitantly stimulating the antifibrotic neprilysin inhibition. Renoprotective evidence is supposed, but evidence is still lacking.

#### 2.2.2. cGMP Effectors

##### cGMP Dependent Protein Kinases (PKG)

The major downstream effector of cGMP is PKG. Two isoforms of PKG are known—PKG1 and PKG2. However, only PKG1 is studied regarding the involvement in renal fibrosis.

Using PKG1-KO mice, Schinner *et al.* [92,119] showed that cGMP acts antifibrotically via activation of PKG1 in UUO. Thereby, the antifibrotic effects of cGMP/PKG1 were mediated by inhibition of the profibrotic RhoA/ROCK signalling to inhibit TGFβ signalling and myofibroblast formation (see above).

In human mesangial cells (HMCs), TWEAK, which is an inflammatory cytokine, led to increased TGFβ expression by stimulation of Ras/Erk1/2. Interestingly, PKG1 protein expression and activity was reduced. Thereby, the Ras/Erk1/2 pathway was essential for the downregulation of PKG1 by TWEAK. Stimulation of PKG1 via 8Br-cGMP abolished TWEAK induced upregulation of TGFβ [177]. These results are in accordance with a study of Cui *et al.*, that increasing PKG activity reduces ECM accumulation in renal mesangial cells. PKG can be activated by increased cGMP levels. In addition to pharmacological approach, PKG transgenic mice (Tg mice) were used to demonstrate the antifibrotic effects of PKG. Tg mice, which expressed more PKG1 than WT mice, showed the same results compared to the treatment with PDE5 inhibitor sildenafil [132]. Until today, no direct PKG activators are in clinical trial, only cGMP elevating agents are being tested.

#### 2.2.3. Further cGMP Influencing Systems

##### Renin Angiotensin Aldosterone System (RAAS)

Angiotensin acts via angiotensin II type 1 receptor (AT_1_R) and angiotensin II type 2 receptor (AT_2_R). Interestingly, AT_2_R has opposite effects of AT_1_R which induces fibrosis. The expression of AT_2_R is increased in pathological situations and is able to suppress cardiac fibrosis. Thereby, the AT_2_R effect is mediated via bradykinin/NO/cGMP pathway AT_2_R thereby forms a heterodimer with the bradykinin receptor B_2_R to induce eNOS which subsequently activates NO/cGMP signalling [178]. The effect of AT_2_R might be promising but is not yet explored in the kidney.

Diabetes mellitus is accompanied by an increase of the RAAS activity, including ATII and aldosterone production. Increased aldosterone was associated with reduced NO/cGMP and increased fibrosis in diabetic kidney [10]. The renin inhibitor aliskiren blocked ATII and aldosterone production [179] and the dihydropyridine-type calcium channel blocker amlodipine reduced aldosterone levels [180]. Decrease of aldosterone and its downstream effectors via aliskiren, amlodipine or the combination of both lead to reduced oxdidative stress and fibrosis via enhanced NO/cGMP availability in the kidney [10].

##### Kallikrein

Bradykinin mediates vasodilatory and antiinflammatory action through NO signalling. Furthermore, kallikrein positively modulates bradykinin production which subsequently activates eNOS. This mechanism is responsible, among others, for its antifibrotic effects.

Kallikrein reverses salt-induced renal fibrosis and glomerular hypertrophy in the interstitium and vasculature of hypertensive Dahl salt-sensitive rats [181]. The repair of renal tubular damage was also observed in a gentamycin-induced nephrotoxicity in normotensive rats [182]. Similar results were shown in a mineralocorticoid-induced renal fibrosis [183].

##### All-Trans-Retinoic Acid/Sodium Butyrate

All-trans retinoic acid in combination with sodium butyrate showed synergistical effects in reducing renal fibrotic biomarkers by enhancing *Npr1* gene transcription which encodes for the GC-A/NPR-A [184]. Renal fibrosis and immunoexpression of renal α-SMA was reduced by ≥70%, and TNFα as well as IL-6 showed lower plasma and renal levels. This could be an important finding for the prevention of hypertension-related kidney diseases.

## 3. Conclusions

Cyclic nucleotide signalling plays a prominent role in the development as well as in the prevention or amelioration of progressive renal disease. cGMP concentrations are diminished during kidney fibrosis. Enhancement of cyclic nucleotides improves renal fibrosis at different stages. They reduce TGFβ signalling, transcription of profibrotic cytokines, oxidative stress, myofibroblast formation and subsequently ECM accumulation in different experimental models for studying renal fibrosis *in vitro* and *in vivo*. Therapeutic approaches are in development, but treatment options modulating cyclic nucleotides are still lacking in clinical practice. Hence, preclinical experimental research is indispensable for the understanding of cyclic nucleotide dependent antifibrotic signalling and for the generation of drugs that may find their way into clinical practice for the treatment or prevention of kidney failure.
